# Rapid community point-of-care testing for COVID-19 (RAPTOR-C19): protocol for a platform diagnostic study

**DOI:** 10.1186/s41512-021-00093-8

**Published:** 2021-02-08

**Authors:** Brian D. Nicholson, Gail Hayward, Philip J. Turner, Joseph J. Lee, Alexandra Deeks, Mary Logan, Abigail Moore, Anna Seeley, Thomas Fanshawe, Jason Oke, Constantinos Koshiaris, James P. Sheppard, Uy Hoang, Vaishnavi Parimalanathan, George Edwards, Harshana Liyange, Julian Sherlock, Rachel Byford, Maria Zambon, Joanna Ellis, Jamie Lopez Bernal, Gayatri Amirthalingam, Ezra Linley, Ray Borrow, Gary Howsam, Sophie Baines, Filipa Ferreira, Simon de Lusignan, Rafael Perera, F. D. Richard Hobbs

**Affiliations:** 1 Nuffield Department of Primary Care Health Sciences, University of Oxford, Radcliffe Observatory Quarter, Woodstock Road, Oxford, OX2 6GG UK; 2 National Infection Service, Public Health England, London, UK; 3 Royal College of General Practitioners, 30 Euston Square, London, NW1 2FB UK

**Keywords:** Diagnostic tests, Point-of-care tests, Primary care, Community testing, COVID-19

## Abstract

**Background:**

The aim of RApid community Point-of-care Testing fOR COVID-19 (RAPTOR-C19) is to assess the diagnostic accuracy of multiple current and emerging point-of-care tests (POCTs) for active and past SARS-CoV2 infection in the community setting. RAPTOR-C19 will provide the community testbed to the COVID-19 National DiagnOstic Research and Evaluation Platform (CONDOR).

**Methods:**

RAPTOR-C19 incorporates a series of prospective observational parallel diagnostic accuracy studies of SARS-CoV2 POCTs against laboratory and composite reference standards in patients with suspected current or past SARS-CoV2 infection attending community settings. Adults and children with suspected current SARS-CoV2 infection who are having an oropharyngeal/nasopharyngeal (OP/NP) swab for laboratory SARS-CoV2 reverse transcriptase Digital/Real-Time Polymerase Chain Reaction (d/rRT-PCR) as part of clinical care or community-based testing will be invited to participate. Adults (*≥* 16 years) with suspected past symptomatic infection will also be recruited. Asymptomatic individuals will not be eligible. At the baseline visit, all participants will be asked to submit samples for at least one candidate point-of-care test (POCT) being evaluated (index test/s) as well as an OP/NP swab for laboratory SARS-CoV2 RT-PCR performed by Public Health England (PHE) (reference standard for current infection). Adults will also be asked for a blood sample for laboratory SARS-CoV-2 antibody testing by PHE (reference standard for past infection), where feasible adults will be invited to attend a second visit at 28 days for repeat antibody testing. Additional study data (e.g. demographics, symptoms, observations, household contacts) will be captured electronically. Sensitivity, specificity, positive, and negative predictive values for each POCT will be calculated with exact 95% confidence intervals when compared to the reference standard. POCTs will also be compared to composite reference standards constructed using paired antibody test results, patient reported outcomes, linked electronic health records for outcomes related to COVID-19 such as hospitalisation or death, and other test results.

**Discussion:**

High-performing POCTs for community use could be transformational. Real-time results could lead to personal and public health impacts such as reducing onward household transmission of SARS-CoV2 infection, improving surveillance of health and social care staff, contributing to accurate prevalence estimates, and understanding of SARS-CoV2 transmission dynamics in the population. In contrast, poorly performing POCTs could have negative effects, so it is necessary to undertake community-based diagnostic accuracy evaluations before rolling these out.

**Trial registration:**

ISRCTN, ISRCTN14226970

**Supplementary Information:**

The online version contains supplementary material available at 10.1186/s41512-021-00093-8.

## Background

The world is in the midst of the SARS-CoV-2 (COVID-19) pandemic. Strict social distancing policies were introduced in many countries to suppress the first wave of infection with negative socio-economic consequences and widespread disruption to healthcare provisio n[1, 2]. Public health policy has focused on rapid identification and isolation (‘test and trace’ [3]) of new cases with targeted local responses to control resurgence of new infections, including areas previously deemed to be virus free [4]. However, efforts have been limited by both shortfalls in laboratory infrastructure, skilled human resources and testing reagents [5], delays in receiving results, and tracing subsequent contacts of positive cases [6]. Consequentially policy and guidance remain blunt, with delayed identification of localised outbreaks in the UK [7], sudden closure of travel corridors to Europe [8], and ongoing concerns about the feasibility of re-opening schools for the forthcoming academic year [9].

Point-of-care tests (POCTs) provide rapid results allowing people to receive immediate advice about self-isolation and treatment, reducing delays and costs associated with sample transportation and reliance upon specialised laboratories [10]. Additional public health impacts of rapid results include reducing residential transmission of infections and improving surveillance of health and social care staff. Whilst there has been accelerated development of POCTs for SARS-CoV-2, there is a lack of data on test safety, accuracy, and utility and performance [11]. Point-of-care antibody testing is well-established in other infections and is easily scalable [12], but initial studies in SARS-CoV-2 have had disappointing results [13]. Novel molecular technologies remain unproven in this context [14]. Accordingly, experts in diagnostics ward against widespread use of POCTs for SARS-CoV-2 before in-context evaluation [11, 15], and medical regulators require clear evidence of acceptable sensitivity and specificity prior to approval [16].

In-context evaluation must reflect both the dynamics of disease transmission and the capabilities of those performing the test to be confident about the test performance in a particular setting. Most of the current data for analytical performance of new POCTs for SARS-CoV-2 is from small and selective patient samples tested within laboratories by highly trained staff [17–19]. Studies in clinical settings have focused on hospitalised patients who are more likely to have higher viral loads, and may undergo invasive procedures to increase yield of respiratory tract sampling [20]. Community settings are characteristically different, and extrapolating results from elsewhere risks spectrum bias and low confidence in results [21]. POCTs must work well when there is a lower prevalence and severity of disease, overlap in presentation with other common clinical syndromes, and in elderly and frail patients who may mount weaker immune responses [22]. Community staff performing POCTs have little-or-no laboratory experience and no ready access to technical support. False negatives are more damaging as ambulatory patients can potentially propel community transmission, whilst false positives in otherwise healthy individuals could hamper efforts to kickstart return to work and education [23].

We therefore aim to undertake context-specific evaluations of multiple POCTs, across a national network of community settings. Our platform design will allow for flexibility in which POCTs are evaluated, changes in Public Health England (PHE) choice of reference standard, and dynamics of local infection rates. RApid community Point-of-care Testing fOR COVID-19 (RAPTOR-C19) is the community testbed to the COVID-19 National DiagnOstic Research and Evaluation Platform (CONDOR) [24]. CONDOR co-ordinates evaluation of diagnostic performance of in vitro diagnostics, across laboratory, community, interface medicine, and secondary-care networks.

This document summarises the key points of the RAPTOR-C19 diagnostic study protocol.

## Methods

### Aim

To assess the diagnostic accuracy of multiple current and emerging POCTs for active or past SARS-CoV-2 infection in the community setting

### Target condition

Active or past COVID-19 (symptomatic disease)

### Primary objective

- Assess the standard diagnostic accuracy of POCTs for active SARS-CoV-2 infection compared to the Public Health England (PHE) reference laboratory standard or equivalent

### Secondary objectives

- Assess the standard diagnostic accuracy of POCTs for past SARS-CoV-2 infection compared to the PHE laboratory reference standard
- Assess the diagnostic accuracy of POCTs for active SARS-CoV-2 infection against an enhanced composite reference standard using multiple tests data, linked electronic health records (EHR) data, and patient reported outcomes data
- Assess the diagnostic accuracy of POCTs for past SARS-CoV-2 infection against an enhanced composite reference standard using multiple tests data, linked EHRs, and patient reported outcomes data

### Study design

RAPTOR-C19 will incorporate a series of prospective observational parallel diagnostic accuracy studies of SARS-CoV-2 POCTs against laboratory and composite reference standards in patients with suspected current or past COVID-19 attending community settings such as general practice. Because the current reference tests are imperfect, the RAPTOR-C19 protocol allows ‘standard’ and ‘enhanced’ diagnostic accuracy studies for active and past infection. As the study is observational in nature, point-of-care test (POCT) results will not be shared with the participant or used to make any clinical decisions.

### Participant selection

Both adults and children will be eligible for participation in the study. The inclusion criteria, listed in Table 1, are broad and designed to capture all-comers to general practice [25]. The clinical presentation of COVID-19 is heterogenous and still being characterised [26]. There are no reliable symptoms to discriminate severity of disease [27]. Restricting testing to a narrow spectrum of clinical features would therefore be inappropriate.

**Table 1:** Inclusion and exclusion criteria

**Inclusion criteria** 1. Adults (> 16 years) (a) Male or female (b) With suspected current or past COVID-19 (symptomatic SARS-CoV2 infection) (c) Having OP/NP swab for laboratory SARS-CoV2 RT-PCR as part of clinical care/testing (d) Willing and able to give informed consent for participation in the study 2. Children (< 16 years old) (a) Male or female (b) With suspected current or past COVID-19 (symptomatic SARS-CoV2 infection) (c) Having OP/NP swab for laboratory SARS-CoV2 RT-PCR as part of clinical care/testing (d) Parent or legal guardian is willing and able to give informed consent for participation in the study **Exclusion criteria** 1. The participant may not enter the study if any of the following apply: (a) Adults unable to understand the study information and give consent to take part in the study (b) Need for immediate hospitalisation (c) Previously enrolled in this study in relation to the individual test being evaluated (d) Asymptomatic	

In general practice settings, the diagnosis of suspected current or past COVID-19 will be based on the clinical judgement of the primary care practitioner and/or the account of the participant. In all community settings, the clinical characteristics of the participant and reasons for testing will be documented.

The working definition of suspected current or past COVID-19 will be based on national advisory guidance [28, 29] to consider SARS-CoV-2 infection in people who, during the current pandemic, have the following:

1. Symptoms thought to be associated with COVID-19, including but not limited to: fever, cough, fatigue, dyspnoea, sputum production, anosmia, change in sense of taste, shortness of breath, myalgia, chills, dizziness, headache, sore throat, hoarseness, nausea, vomiting, diarrhoea, nasal congestion
2. Acute respiratory distress syndrome
3. Either clinical or radiological evidence of pneumonia
4. Atypical presentations, for example an acute functional decline or frailty syndrome in an older person, if they are immunocompromised
5. Symptoms and lived or worked in close contact with somebody who has tested positive for SARS-CoV-2, including NHS staff

### Setting

RAPTOR-C19 sites will be community based. The primary community setting will be primary care, focussing on general practices reviewing patients with suspected COVID-19 or acting as COVID-19 hubs. Practices that have submitted an expression of interest to take part in the study will be selected with the help of the National Institute for Health Research (NIHR) Clinical Research Network (CRN). RAPTOR-C19 sites will be reimbursed per patient recruited for their participation in the research. Participants will not be paid for their participation in the research.

Practices will be initially recruited through Oxford Royal College of General Practitioners (RCGP) Clinical Informatics Digital Hub (ORCHID) [30]. The surveillance platform of ORCHID is called the Oxford-RCGP Research and Surveillance Centre (RSC). General practices within this network are experienced in taking virology samples and serology specimens and have adapted their long established influenza surveillance to provide a sentinel system for COVID-19, including sero-surveillance [31]. Additionally, they have also previously successfully integrated POCTs into primary care clinical workflow in the 2018/2019 flu season [32, 33] with an additional pilot conducted across the 2019/2020 season. Recruiting patients into RAPTOR-C19 is an integral part of the planned 2020/21 ORCHID/PHE surveillance [34]. Patients who volunteer to provide samples for surveillance will have the opportunity to take part in RAPTOR-C19 in participating practices, and samples taken, with consent through RAPTOR-C19, will also be available to use for national surveillance.

### Eligibility assessment and recruitment

There are two routes to potential participants assessed for eligibility: opportunistic and remote.

Opportunistic recruitment is the primary recruitment strategy. This follows a patient-initiated contact with the RAPTOR-C19 study site, with symptoms consistent with current COVID-19 or past symptoms of possible/confirmed COVID-19.

Remote assessment represents a potential secondary recruitment strategy to be used if the primary recruitment strategy is not effective. The electronic health record (EHR) will be searched to identify people who have presented with symptoms of possible COVID-19, have had a confirmed diagnosis, or are a symptomatic household contact of a confirmed case. These people could then be invited for assessment of eligibility.

Asymptomatic individuals are not eligible.

### Informed consent

The RAPTOR-C19 site team will ask eligible and willing patients (or their parent/carer, where applicable) to complete an e-consent process (Supplementary Figure S1). Informed consent will be obtained in line with Good Clinical Practice (GCP) guidelines.

It is imperative that all non-essential contact between the participants, researchers, and practice staff is prevented in order to minimise the risk of SARS-CoV-2 transmission. To achieve this, we will use a combination of digital written consent and/or researcher recorded verbal consent in this study, in person, or remotely by telephone or using video link. Written information will be available in the form of posters at RAPTOR-C19 sites, and as electronic participant information accessible online at https://www.condor-platform.org/condor_workstreams/raptor (Supplementary Figure S2).

The participant will be allowed as much time as wished to consider the information, and the opportunity to question the researcher or other independent parties to decide whether they will participate in the study. All answers will be stored electronically and securely.

### Data collection

RAPTOR-C19 have developed a bespoke data collection solution with uMed, a UK based health-technology company. Through a series of secure webpages, the uMed platform will allow the participant, or the researcher on behalf of the participant, to record eligibility and to document consent. The uMed platform will guide the participant, or the participant’s parents/guardians, through the consent questions, or the researcher will read out the questions from the form, recording the participant’s responses electronically. The completed consent form will be exported into a pdf document and emailed to the participant.

Consenting participants will be asked for further study-specific information, which will be entered into the electronic case report form (eCRF). RAPTOR-C19 will provide study sites with a Wi-Fi- and 4G-enabled tablet to collect study data. However, it will be possible to assess eligibility and to gather consent and additional participant information using any internet enabled device.

ORCHID practices currently share pseudonymised data for surveillance and research twice weekly, with practices migrating to a daily extract. ORCHID data is also linked to hospital and death data to allow robust identification of outcomes. Individual patients volunteering to participate in RAPTOR can have relevant health data linked to the study database.

### Baseline assessments

For adults (≥ 16 years old) study visits will follow the same protocol whether current or past COVID-19 is suspected: the analysis will be different. In children (< 16 years), only those with suspected current COVID-19 will be included. In all cases, the baseline visit will involve the POCT(s) under evaluation and the tests for laboratory reference testing (see below). Following consent being provided, the eCRF will then be used to capture study data as detailed in Supplementary Box S1.

### Index tests and reference standards

#### Index tests (POCTs)

Biological samples to test for current SARS-CoV-2 infection will be collected from all participants. The index test will be at least one, but the intention is to assess multiple, candidate POCTs for active infection (all participants) or past infection (adults only). If multiple POCTs are being assessed, these may target a combination of current SARS-CoV-2 infection and past SARS-CoV-2 infection.

Participants will be asked to submit samples as appropriate for each candidate POCT by following the training and instructions provided by the manufacturer. These may include oropharyngeal/nasopharyngeal (OP/NP) swab, saliva, or blood from a finger prick. POCTs requiring finger prick blood samples will only be offered to participants > 10 years old. For POCTs that require assistance to complete, the researcher will assist the participant whilst adhering to safe PPE use. Where a participant completes the tests themselves, they will be observed by the researcher to monitor correct POCT use and ease of use and to identify sample quality issues.

The order in which the tests are conducted will not be randomised but the sequencing of the tests will be documented in the eCRF. Who performs the test, either participant or researcher, will also be recorded.

All POCT consumables will be discarded as clinical waste as soon as the POCT is complete and the results have been captured. No POCT samples will be retained by the RAPTOR-C19 team.

Both the researcher and the participant will be blinded from results of the reference test, as results will not be returned for at least 24 h after the POCT is taken. For paired index tests blinding will not be possible in a clinical environment. For qualitative index tests, we will capture a photograph of the result, for adjudication by independent research staff, blinded to any other clinical information or test results. POCT results will not be shared with the patient and they must not be used to make any clinical decisions.

POCT-specific amendments to the ethical permission and protocol will be completed prior to including each POCT in the assessment. RAPTOR-C19 staff will develop training materials using the manufacturer’s instructions (these will be edited if deemed necessary by the RAPTOR-C19 and the Patient and Public Involvement group). RAPTOR-C19 staff will liaise with the manufacturers where clarification is required on use of the POCT. They will arrange training via teleconference with local study leads and community staff to allow rapid dissemination in compliance with social distancing advice. Online tutorials and/or YouTube videos will be made available. These will be updated as necessary, as new POCTs are introduced into the study. During the study, RAPTOR-C19 staff will be available to support study sites and answer any queries.

#### Reference laboratory tests

The current PHE reference standard for active infection is an OP/NP swab for laboratory real-time RT-PCR triplex assay for the detection of SARS-CoV-2. It incorporates multiplex detection of two SARS-CoV-2 targets (Orf1ab and E genes) and uses a soil borne cereal mosaic virus internal control [35]. It is important to note that reference laboratory tests for current infection will be done as part of clinical care or as part of the national surveillance system. Individuals can have these done whether or not they agree to be part of research.

Only adult participants (≥16 years old) will be asked to submit a blood sample for antibody testing for use as the reference standard for past SARS-CoV-2 infection and within the composite reference standard (see below) for current infection. Adults will have blood samples for this drawn at baseline and follow-up visits by appropriately trained staff.

Participants will receive clear instructions on how to sample, as per PHE standard advice. If participants are unable to self-swab, or express a preference, a staff member with appropriate training will take the sample. The sample material will fall under PHE or other central testing laboratory and not our study remit, and under current rules PHE may retain the swab for up to 5 years. Participants will be able to discuss the results of the PHE reference standards with their GP.

Once taken, the samples will be put in the regulation container packaging, double bagged, and sent to the PHE laboratory or other central testing centre laboratory that is supporting the study using their existing, safe, quality compliant processes. Participants will have the option to agree to this sample being retained for future surveillance or research use.

We acknowledge that the PHE reference standard and the details of the assay may change throughout the study as more accurate reference tests are adopted. POCTs will always be benchmarked against the current best practice. We will report the reference standard tests used in each POCT evaluation when each evaluation is submitted for publication. We will also compare POCTs to any change reference standards to mitigate the imperfect reference tests, and adjust our statistical analysis to reflect these potential changes.

### Subsequent visits

Where feasible, adult (≥ 16) participants will also be invited to attend a second visit, or visited at home by a research nurse, 28 days following the first visit, to allow for a blood test for repeat antibody testing as outlined above.

Adult participants may be contacted by text message to complete an online daily symptom diary prior to the follow-up visit.

The time schedule of enrolment, assessments, and visits for participants is summarised in Table 2.

**Table 2:**
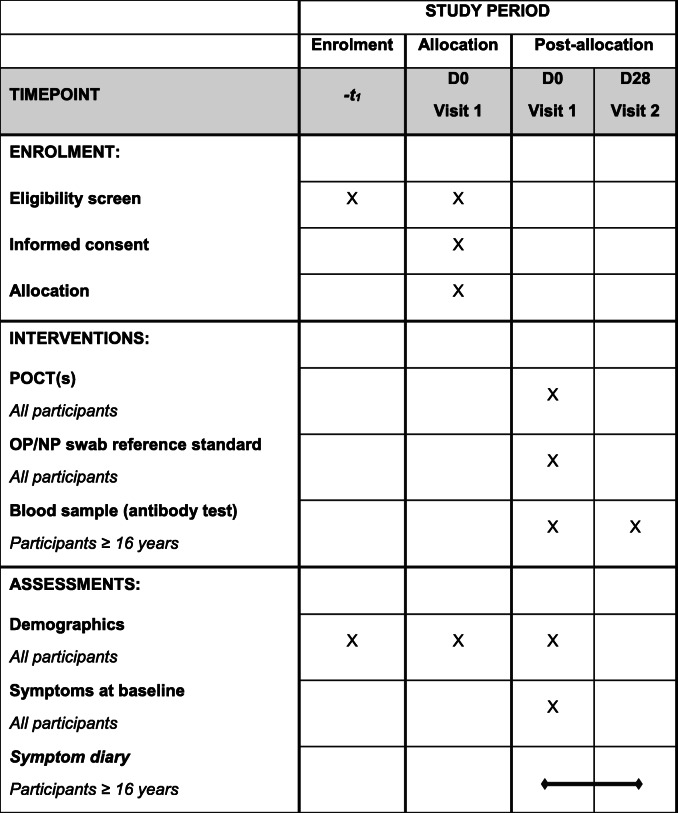
Schedule of enrolment, interventions, and assessments for RAPTOR-C19

### Study end

Recruitment will be reviewed by the Study Management Committee, using the latest prevalence data from PHE, as prevalence of SARS-CoV-2 is dynamic and affects the sample size required. Each participant has the right to withdraw from the study at any time. Withdrawn participants will not be replaced. Participants are not required to give a reason for withdrawal. The investigators may discontinue a participant from the study at any time if they consider it necessary for any reason including the following:

- Ineligibility (either arising during the study or retrospectively having been overlooked at eligibility assessment)
- Significant protocol deviation
- Withdrawal of consent
- If the participant declines POCTs, or if an adult (≥ 16 years) declines to give a venous blood sample

### Safety

All RAPTOR-C19 sites will be required to follow the current PHE infection prevention and control guidance regarding collection and processing of samples at all times including that regarding PPE. Contact will be minimised by using electronic and/or verbal consent, where possible remotely by telephone or video calls.

Safety reporting is not applicable given the low risk of point-of-care tests. Nose and throat swabs cause some transient discomfort to patients, but there are no clinically significant risks associated with the procedure. Fingerstick blood sampling may cause transient discomfort and localised bruising at the sampling site; however, there are no clinically significant risks associated with the procedure. Venous blood sampling causes discomfort and may result in bruising and localised swelling at the sampling site. Provision of saliva samples is unlikely to cause discomfort to any participants. To mitigate these risks, self-sampling will be supported where appropriate, otherwise these procedures will be carried out by personnel who have received training in these procedures or who carry out these procedures as a routine element of their duties.

A serious adverse event is any untoward medical occurrence that does the following:

- Results in death
- Is life-threatening
- Requires inpatient hospitalisation or prolongation of existing hospitalisation
- Results in persistent or significant disability/incapacity
- Consists of a congenital anomaly or birth defect

Serious adverse event (SAE) occurring to a participant will be reported to the REC that gave a favourable opinion of the study where in the opinion of the Chief Investigator the event was ‘related’ (resulted from administration of any of the research procedures) and ‘unexpected’ in relation to those procedures. Reports of related and unexpected SAEs will be submitted within 15 working days of the Chief Investigator becoming aware of the event, using the Health Research Authority (HRA) report of serious adverse event form (see HRA website).

### Statistics and analysis

The statistical aspects of the study are summarised here with details fully described in a statistical analysis plan (SAP). The SAP will be finalised before any analysis takes place.

### Data sources

Table 3 outlines which data sources used to address each research question.

**Table 3:** Data sources for analysis

Question	Data source					
	eCRF	*POCT index test* for *active* COVID-19	*Laboratory reference test* for *active* COVID-19	*POCT index test* for *past* COVID-19	*Laboratory reference test* for *past* COVID-19	*Composite reference standard*
*Standard* diagnostic accuracy of *active* infection	Yes	Yes—visit 1. Current and past suspects	Yes—visit 1. Current and past suspects	No	No	No
*Enhanced* diagnostic accuracy of *active* infection	Yes	Yes—visit 1. Current and past suspects	Yes—visit 1. Current and past suspects	No	Yes—visit 2. Current and past suspects	Yes
*Standard* diagnostic accuracy of *past* infection	Yes	No	No	Yes—visit 1. Current and past suspects	Yes—visit 1. Current and past suspects	No
*Enhanced* diagnostic accuracy of *past* infection	Yes	Yes—visit 1. Current and past suspects	Yes—visit 1. Current and past suspects	Yes—visit 1. Current and past suspects	Yes—visit 1 and 2. Current and past suspects	Yes

### Composite reference standards

An assumption of standard diagnostic accuracy studies is that the reference standard is infallible. This constrains the performance of the index test to the performance of the reference standard and assumes every time the tests get different results the reference is correct and the index is incorrect. There is evidence of substantial heterogeneity of diagnostic accuracy of SARS-CoV-2 d/rRT-PCR testing, with reported false negative rates of 18–58% [36]. Both clinical context and repeated testing are highly relevant to calculating the likelihood of true negative cases [37], and so we will undertake further analyses using the composite reference standard.

Composite reference standard 1 will be designed to minimise false negatives (FNs), and composite reference standard 2 will be designed minimise false positives (FPs). Both composite reference standards will be constructed considering paired antibody test results, patient reported outcomes, linked EHRs for outcomes related to COVID-19, such as hospitalisation or death, other test results (Table 4).

**Table 4:** Potential use of tests to enhance the reference standard. Information in the table refers to hypothetical combinations of results in which the enhanced reference standard result would be considered different from the original reference standard result. A cell-by-cell interpretation of each scenario follows.

	Minimise FN for current infection		Minimise FN for past infection	
Visit (day)	1 (0)	2 (28)	1 (0)	2 (28)
**SARS-CoV-2 RT-PCR**	Negative (FN)	N/A	Negative	N/A
**COVID IgG**	Negative	Positive	Negative (FN)	Positive
Or:	Subsequent positive SARS-Cov-2 RT-PCR result (within 28 days)		-	
Or:	Hospital admission or death (within 28 days) citing SARS-CoV-2 as probable cause		Hospital admission or death (within 28 days) citing SARS-CoV-2 as probable cause	
	**Minimise FP for current infection**		**Minimise FP for past infection**	
**Visit (day)**	1 (day 0)	2 (28)	1 (day 0)	2 (28)
**SARS-CoV-2 RT-PCR**	Positive (FP)	N/A	Negative	N/A
1.1.35. **COVID IgG**	Negative	Negative	Positive (FP)	Negative
And:	No subsequent positive SARS-Cov-2 RT-PCR result (within 28 days)		-	
And:	No hospital admission or death (within 28 days) citing SARS-CoV-2 as probable cause		No hospital admission or death (within 28 days) citing SARS-CoV-2 as probable cause	

Minimise FN (false negative) for current infection: If the COVID-19 RT-PCR reference test gives a negative result, it can be considered a false negative if the IgG result is positive at 28 days (newly positive or titres have increased), OR if a subsequent SARS-Cov-2 RT-PCR result (within 28 days) was observed, OR if there was a hospital admission or death (within 28 days) citing SARS-CoV-2 as probable cause

Minimise FP (false positive) for current infection: If the COVID-19 RT-PCR reference test gives a positive result, it can be considered a false positive if the IgG result is negative at both baseline and 28 days, AND if no subsequent SARS-Cov-2 RT-PCR result (within 28 days) was observed, AND if there was no hospital admission or death (within 28 days) citing SARS-CoV-2 as probable cause

Minimise FN (false negative) for past infection: If the baseline reference IgG test result is negative, it can be considered a false negative if the 28-day IgG test result is positive, OR if there was a hospital admission or death (within 28 days) citing SARS-CoV-2 as probable cause

Minimise FP (false positive) for past infection: If the baseline reference IgG test result is positive, it can be considered a false positive if all other tests at baseline and at 28 days are negative, AND there has been no hospital admission or death (within 28 days) citing SARS-CoV-2 as probable cause

Paired antibody testing at 0 and 4 weeks will identify changes in antibody levels over time. These serial laboratory samples will allow us to identify antibody changes to discriminate between current and past infection, and to highlight possible FP and FN RT-PCRs, whilst it remains possible that infection could occur after the index test but prior to the day 28 serology sample, this is unlikely to be a frequent event.

### Statistical analysis

Results will be presented according to the Standards for Reporting Diagnostic accuracy studies (STARD) guidelines for reporting diagnostic studies.

### Descriptive analysis

Characteristics of recruited participants will be summarised using tables and graphs. If applicable, these will be compared to estimates from the general population. Number of total valid tests by POCT and reference standards will also be reported (actual and percentages), stratified by children vs adults and by age groups (if feasible dependent on total counts).

### Summary statistics of diagnostic accuracy

Sensitivity, specificity, and positive and negative predictive values for each POCT will be calculated with exact 95% confidence intervals.

For the primary outcome and first secondary outcome:

For consecutive POCTs for active infection, the diagnostic accuracy of each POCT will be summarised independently using 2 × 2 tables for POCT (+/-) and the current standard PHE reference test (+/-) for active infection. For consecutive POCTs for past infection, the diagnostic accuracy of each POCT will be summarised independently using 2 × 2 tables for POCT (+/-) and the current standard PHE reference test (+/-) for past infection. Measures of diagnostic accuracy will also be presented adjusted for imperfect reference standard bias, using the best available estimate of the accuracy of the PHE reference test.

For the second and third secondary outcomes:

For consecutive POCTs for active infection, the enhanced diagnostic accuracy of each POCT will be summarised independently using 2 × 2 tables for POCT (+/-) and the composite reference standards as outlined in Table 4 (+/-). For consecutive POCTs for past infection, the enhanced diagnostic accuracy of each POCT will be summarised independently using 2 × 2 tables for POCT (+/-) and the composite reference standards (+/-) for past infection.

Results for the primary outcome will be stratified by adults vs children, by age group (< 16, 16–40, 40–60, 60+ years), by gender, by ethnicity and by spectrum of disease (a combined measure of symptom severity and duration). A further subgroup analysis will test for differences in diagnostic performance by practice, and if any differences are detected, associations with practice size, location and model of care will be explored.

### Missing data

Missing data for test results including reference tests will be reported. Potential associations between patient characteristics (e.g. age, gender) and the pattern of missing data will be evaluated and reported using tables and graphs. Robustness of the estimates for accuracy will be evaluated using sensitivity analyses.

### Number of participants

We have calculated sample sizes using standard methodology based on minimum clinically relevant sensitivity or specificity (whichever is the most critical for the intended placement in the care pathway), not expected values from preliminary work [16]. For example, acceptable thresholds for minimum sensitivity and specificity of 80% and 95% respectively, can be used to determine sample size requirements and a strategy for early identification of poorly performing tests.

### Stopping criteria

Assuming a test with 90% sensitivity, a 99% specificity, and a pre-test probability (prevalence) of 30%, we would require 200 participants to meet the minimum thresholds as stated above. This would also mean that tests with more than seven false negatives or two false positives could be immediately dropped from the study. This allows us to exclude tests with sensitivities of 50%, 60%, 70%, or 80% after the first 50, 60, 80, and 120 participants recruited. For tests with poor specificities of 80%, 85%, 90%, or 95% these would be identified after 15, 20, 30, and 60 participants recruited. The prevalence of COVID-19 affects the sample size required. With a pre-test probability (prevalence) of 5% we would require 1200 participants to evaluate a POCT and interim futility analyses will stop the evaluation after seven false negatives or twelve false positives. POCTs with an actual sensitivity of 50%, 60%, 70%, or 80% would reach futility criteria after the first 280, 350, 470, and 700 participants. POCTs with an actual specificity of 80%, 85%, 90% or 95% would reach futility criteria after the first 65, 85, 130, and 255 recruited participants.

Evaluations of the first POCTs will be performed without implementation of stopping criteria to establish whether sufficient information to create the composite reference standard can be obtained sufficiently quickly to make these criteria viable. The figures presented here should therefore be regarded as indicative for a POCT designed to detect active infection and will be reviewed once this information becomes available.

### Illustrative sample sizes

Table 5 presents illustrative sample sizes to achieve a range of POCT sensitivities based on a standard error of 2.5%. A standard error of 2.5% will give a confidence interval of 5% on either side of the sensitivity estimate.

**Table 5:** Illustrative sample sizes to achieve a range of POCT sensitivities based on a standard error of 2.5%

	Prevalence	40%	35%	30%	25%	20%	15%	10%	5%
**Sensitivity**	**95%**	190	218	254	304	380	507	760	1520
	**90%**	360	412	480	576	720	960	1440	2880
	**85%**	510	583	680	816	1020	1360	2040	4080
	**80%**	640	732	854	1024	1280	1707	2560	5120
	**75%**	750	858	1000	1200	1500	2000	3000	6000
	**70%**	840	960	1120	1344	1680	2240	3360	6720

### Data management, governance, and data access

The University of Oxford is the sponsor of this research. The study will comply with the General Data Protection Regulation (GDPR) and Data Protection Act 2018. The data management policy and governance framework can be found at https://www.condor-platform.org/condor_workstreams/raptor. Only substantive employees of the University of Oxford will have access to the data and only for the purposes described in the study protocol. Direct access will be granted to authorised representatives from the sponsor and host institution for monitoring and/or audit of the study to ensure compliance with regulations. The legal basis for the Oxford-RCGP RSC surveillance is that this is classified as Health Protection under Regulation 3 of The Health Service (Control of Patient Information) Regulations 2002 and approved annually by the Public Health England Caldicott Guardian; other studies require appropriate ethical approval.

### Dissemination and publication policy

We will publish the results in open-access journals, the protocol on the study website (https://www.condor-platform.org/condor_workstreams/raptor) and registries, and summary reports which can be made publicly available through, e.g. the websites of the study and of the NIHR Community Healthcare MIC (https://www.community.healthcare.mic.nihr.ac.uk/). We will work with patient and public representatives to ensure that such reports are communicated in an appropriate manner for a lay audience. The Investigators will be involved in reviewing drafts of the manuscripts, abstracts, press releases, and any other publications arising from the study. Authors will acknowledge that the study was funded by UKRI-MRC and any other funding that is secured. Authorship will be determined in accordance with the ICMJE guidelines and other contributors will be acknowledged.

## Discussion

There is a need for diagnostic accuracy studies in the community setting of POCTs for the detection of SARS-CoV-2. It is important to identify high-performing tests to prioritise these for use, and to avoid poorly performing technologies. We have set out in this protocol how we will achieve this in UK community settings. The RAPTOR-C19 diagnostic platform has been developed to support the UK’s Urgent Public Health response to COVID-19. Building RAPTOR-C19 on top of an established surveillance network (RCGP RSC) gives efficiencies, gives advantages for digital data collection, and aids recruitment.

### Strengths and limitations

The prevalence of SARS-CoV-2 in the tested population will be the primary determinant of the rate at which RAPTOR-C19 can evaluate the accuracy of each POCT. The UK had surpassed a peak of SARS-CoV-2 infections at the time this protocol was being prepared for publication, with national easing of lockdown underway. COVID-19 testing capacity in the NHS took time to develop, with initial testing mainly occurring in the hospital setting, later through local COVID-19 ‘hubs’ and then national testing centres. At the height of the first peak, the prevalence of infection was > 30% in those tested [38]. As testing increased outside of the hospital setting, the prevalence of SARS-CoV-2 infections fell, due to a combination of decreasing national SARS-CoV-2 infection prevalence and increased testing in a broader population [39]. Whilst we welcome this reduction in prevalence, it results in larger sample sizes for clinical research.

By recruiting symptomatic people who are clinically suspected to have SARS-CoV2 infection, RAPTOR-C19 will increase the likelihood of identifying positive cases in the community above the background prevalence. To maximise recruitment RAPTOR-C19 will recruit from a base of high-throughput practices, with large practice populations, and/or practices acting as COVID-19 hubs, with wide geographical spread throughout the UK, including urban centres with high population density. Additional practices will be asked to act as stand-by sites where evaluation may begin rapidly in response to local outbreaks of SARS-CoV-2. This combined approach is intended to increase the likelihood of capturing positive cases in symptomatic people above the background national prevalence of SARS-CoV-2. From a public health point of view, correctly ruling-in active SARS-CoV-2 is just as important as ruling it out. Therefore, we need POCTs that are both highly sensitive and highly specific. The CONDOR triage process aims to identify POCTs that align with the MHRA specification based on the available data from the manufacturer or independent analytical analyses. By starting with a rigorous selection process, we reduce the probability of evaluating poorly performing POCTs.

A strength of the RAPTOR-C19 protocol is the stopping criteria to identify POCTs that have already exceeded the maximum number of false positives or false negatives necessary to be able achieve the target sensitivity and specificity. A corresponding weakness is that a test which has focussed on achieving high sensitivity but has low specificity may be disadvantaged in a low prevalence setting, especially if the anticipated prevalence used to set the stopping criteria, was higher than the actual prevalence. In this circumstance, tests may be excluded from the analysis because of accumulating too many false positive results in comparison to the laboratory and composite reference standards. Nevertheless, these stopping criteria reflect MHRA mandated performance criteria. During early evaluations we will establish whether sufficient information to create the composite reference standard can be obtained sufficiently quickly to make stopping criteria viable.

We recognise the challenges of recruitment in a changing policy environment, with variable disease prevalence. GP sites will be recruited from the RCGP Research and Surveillance Centre (RSC) is an internationally renowned source of information, analysis, and interpretation of primary care data [40]. The dataset is nationally representative [41], having only small differences with the national population, which have now been quantified and can be assessed for clinical relevance for specific studies. With twice weekly data extractions, the dataset is one of the most up to date in the UK, and now a platform within ORCHID. ORCHID is in the process of enhancing the frequency of extraction and scope of data linkage. It is already extracting primary care computerised medical records (CMRs) daily and linking with linked hospital data and mortality data. ORCHID will additionally support RAPTOR-C19 by allowing patient follow-up within the RCGP RSC network, and the creation of an enhanced composite reference standard to overcome limitations of laboratory reference tests [30].

However, in the pandemic situation, community testing has been expanded in unexpected and unprecedented ways. In order to respond to the needs of the community and maximise recruitment and coverage of different clinical settings, we may also recruit from other community settings where reference RT-PCR swabs are being or can be collected. These might include national testing centres, home testing systems, surveillance and national telephone triage services, pop-up community laboratories in areas of increasing local prevalence, transport infrastructure and the UK borders, and educational or commercial organisations. People tested at these locations will not necessarily be registered at RCGP RSC practices, and the venues may not be able to undertake venepuncture, so it may not be possible to use the same reference standards as at GP practices. Many of these settings undertake service evaluations that align with government priorities, and we would seek to partner with these organisations. We would aim to establish data sharing agreements for a limited set of de-identified participant and test result data to permit the primary evaluation of POCTs used in these settings against the usual RT-PCR reference standard, and depending on the data they are able to collect, modified enhanced standards.

### Future research

The development of the RAPTOR-C19 platform opens the door to future evaluations of POCTs for other infectious diseases in the community alongside or instead of COVID-19 POCTs. The underlying digital infrastructure of ORCHID provides a rich retrospective linked primary care EHR dataset upon which to build future prospective analyses of POCTs for communicable and non-communicable diseases, such as biomarkers for cancer diagnosis or cardiovascular disease monitoring. The rapid growth of the RCGP RSC to support priority COVID-19 research, such as the PRINCIPLE trial, has created the largest network of GP sites available for interventional and diagnostic research in the UK.

### Conclusion

We have described the protocol for a diagnostic accuracy platform for SARS-CoV-2 POCTs specific to community settings. It leverages existing surveillance, digital, and primary care infrastructure and aims to identify POCTs that meet government specified diagnostic performance levels.

## Supplementary Information

**Additional file 1:.** Supplementary Figure S1. Parental Consent Form. Supplementary Figure S2. Participant information sheet for minors (under 6 years). Supplementary Box S1. Baseline data

## Data Availability

Not applicable.

## Abbreviations

CMRs: Computerised medical records
CONDOR: COVID-19 National DiagnOstic Research and Evaluation Platform
COVID-19: Coronavirus 2019 disease
CRN: Clinical Research Network
eCRF: Electronic case report form
EHR: Electronic health record
FN: False negative
FP: False positive
GCP: Good clinical practice
GDPR: General Data Protection Regulation
GP: General practitioner
HRA: Health Research Authority
ICMJE: International Committee of Medical Journal Editors
IgG: Immunoglobulin G
MIC: NIHR Community Healthcare MedTech and In vitro Diagnostics Co-operative
NHS: National Health Service
NIHR: National Institute for Health Research
OP/NP: Oropharyngeal/nasopharyngeal
ORCHID: Oxford Royal College of General Practitioners Clinical Informatics Hub
PC: Primary care
PHE: Public Health England
POCT: Point-of-care test
RAPTOR-C19: RApid community Point-of-care Testing fOR COVID-19
RCGP RSC: Royal College of General Practitioners Research and Surveillance Network
REC: Research ethics committee
RT-PCR: Real-time polymerase chain reaction
SAP: Statistical analysis plan
SARS-CoV-2: Severe Acute Respiratory Syndrome Coronavirus 2
STARD: Standards for Reporting Diagnostic Accuracy Studies
