# Supplementary material for: Rapid community point-of-care testing for COVID-19 (RAPTOR-C19): protocol for a platform diagnostic study
Source: Diagn Progn Res. 2021 Feb 8;5:4. doi: 10.1186/s41512-021-00093-8 (PMC7868893; doi:10.1186/s41512-021-00093-8)
Supplement: Supplementary file 1 — Additional file 1:. Supplementary Figure S1. Parental Consent Form. Supplementary Figure S2. Participant information sheet for minors (under 6 years). Supplementary Box S1. Baseline data [file 41512_2021_93_MOESM1_ESM.docx]

**Supplementary Data**

**Supplementary Figure S1. Parental Consent Form**


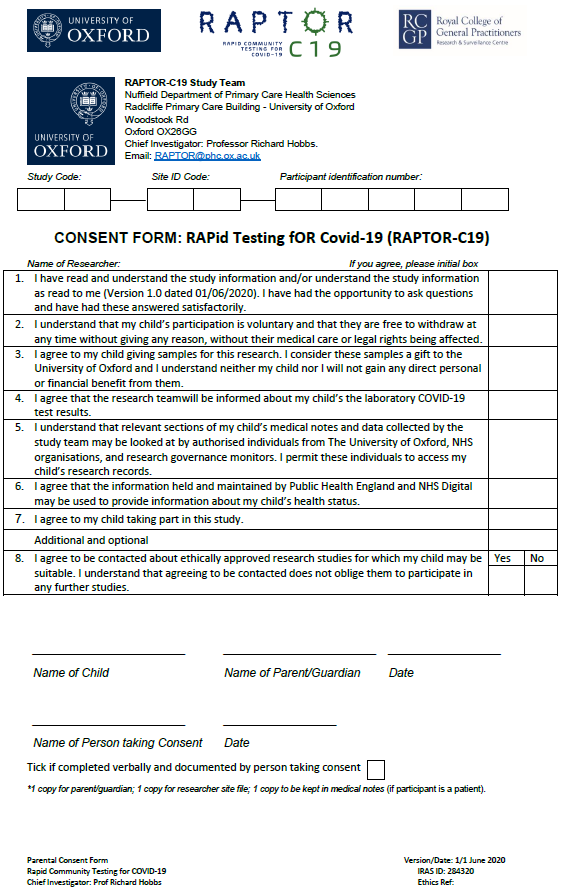


### **Supplementary Figure S2. Participant information sheet for minors (under 6 years)**


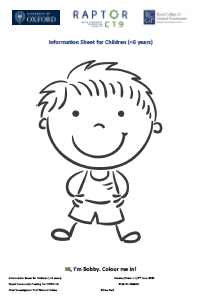

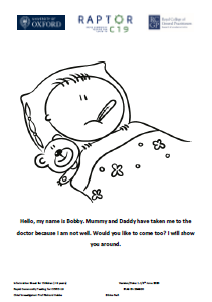

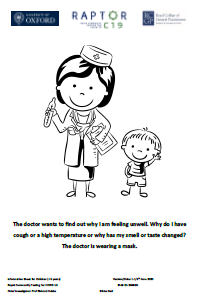

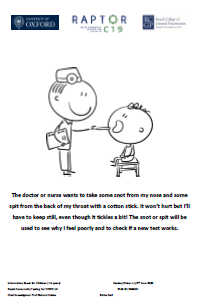

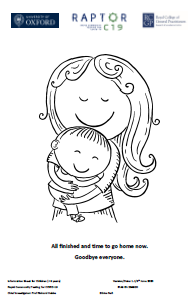


### **Supplementary Box S1. Baseline data**

| 1. **Spectrum of disease data**    1. gender    2. age    3. ethnicity    4. comorbidities    5. current date    6. symptoms    7. duration of symptoms    8. household COVID-19 contacts    9. clinical observations (if available)    10. immediate place of care    11. care home resident    12. vaccine status (experimental or new COVID vaccine)    13. past COVID tests with results 2. **Test data**    1. POCT (repeated subsection if multiples POCTs)       1. POCT for active or past infection       2. Test ID       3. Time of test       4. Who is performing the POCT       5. Results  - a description and photo of qualitative results  - a continuous quantitative result with units of measurement       6. Usability of test (Likert scale)       7. Problems (errors / indeterminate results / not done / failed with reason)    2. COVID-19 reference swab       1. Test completion       2. Time of test       3. Who took the swab    3. Reference antibody blood sample       1. Test completion       2. Time of test       3. Problems with venepuncture    4. Sequencing of tests |
| --- |
